# Supplementary material for: Large scale crowdsourced radiotherapy segmentations across a variety of cancer anatomic sites
Source: Sci Data. 2023 Mar 22;10:161. doi: 10.1038/s41597-023-02062-w (PMC10033824; doi:10.1038/s41597-023-02062-w)
Supplement: Supplementary file 1 — Supplementary Table 1 [file 41597_2023_2062_MOESM1_ESM.docx]

**Supplementary Table 1.** Summary of all region of interest (ROI) segmentations generated by participants for this data descriptor. ROIs included radiotherapy target volumes and organs at risk (OARs).

| **Case** | **Type of ROI** | **ROI** | **Definition(s)** | **Number of expert segmentations** * | **Number of non-expert segmentations** * |
| --- | --- | --- | --- | --- | --- |
| Breast | Target volumes | CTV_Ax | Clinical target volume of axillary region | 8 | 115 |
|  |  | CTV_Chestwall | Clinical target volume of chest wall | 8 | 117 |
|  |  | CTV_IMN | Clinical target volume of internal mammary nodes | 8 | 118 |
|  |  | CTV_Sclav_LN | Clinical target volume of supraclavicular lymph nodes | 8 | 119 |
|  | OARs | BrachialPlex_L | Brachial plexus left | 6 | 88 |
|  |  | Heart | Heart | 7 | 121 |
|  |  | A_LAD | Left anterior descending artery | 7 | 88 |
| Sarcoma | Target volumes | GTV | Gross tumor volume | 5 | 60 |
|  |  | CTV | Clinical tumor volume | 5 | 48 |
|  | OARs | Genitals | Genitalia | 4 | 51 |
| Head and Neck | Target volumes | GTVp | Gross tumor volume primary - right tonsillar fossa | 14 | 59 |
|  |  | GTVn | Gross tumor volume of nodes - nodal spread to level II/III on ipsilateral side (with sternocleidomastoid muscle invaded) and no contralateral nodal involvement | 13 | 60 |
|  |  | CTV1 | Clinical target volume (high-risk) | 9 | 45 |
|  |  | CTV2 | Clinical target volume (low to intermediate risk) | 9 | 49 |
|  | OARs | Brainstem | Brainstem | 13 | 58 |
|  |  | Glnd_Submand_L | Submandibular gland left | 13 | 57 |
|  |  | Glnd_Submand_R | Submandibular gland right | 12 | 52 |
|  |  | Larynx | Larynx | 12 | 57 |
|  |  | Musc_Constrict | All pharyngeal constrictor muscles (superior, middle, and inferior) | 11 | 43 |
|  |  | Parotid_L | Parotid left | 13 | 59 |
|  |  | Parotid_R | Parotid right | 13 | 58 |
| Gynecologic | Target volumes | GTVn | Gross tumor volume of the involved right common iliac lymph node | 5 | 42 |
|  |  | CTVn_4500 | Clinical target volume for the elective nodal volumes at risk that will receive 45 Gy | 5 | 40 |
|  |  | CTVp_4500 | Clinical target volume primary will receive 45 Gy. This is the combination of “ctv1” and “ctv2” used in many RTOG protocols | 5 | 41 |
|  | OARs | Bowel_Small | Small bowel | 4 | 35 |
| Gastrointestinal | Target volumes | CTV_4500 | Clinical target volume that will receive 45 Gy | 4 | 25 |
|  |  | CTV_5400 | Clinical target volume that will receive 54 Gy | 4 | 23 |
|  | OARs | Bag_Bowel | Small and large bowel | 4 | 23 |

* Not all participants generated segmentations for all ROIs.
