# Supplementary material for: Large scale crowdsourced radiotherapy segmentations across a variety of cancer anatomic sites
Source: Sci Data. 2023 Mar 22;10:161. doi: 10.1038/s41597-023-02062-w (PMC10033824; doi:10.1038/s41597-023-02062-w)
Supplement: Supplementary file 2 — Supplementary Table 2 [file 41597_2023_2062_MOESM2_ESM.docx]

**Supplementary Table 2.** Pairwise interobserver variability values for experts and non-experts. Pairwise Dice similarity coefficient (DSC), average surface distance (ASD), and surface DSC (SDSC) are shown for experts and non-experts separately. Median values reported with interquartile range in parenthesis.

| **Case** | **Type of ROI** | **ROI** | **Expert DSC** | **Expert ASD** | **Expert SDSC** | **Non-Expert DSC** | **Non-Expert ASD** | **Non-Expert SDSC** |
| --- | --- | --- | --- | --- | --- | --- | --- | --- |
| Breast | Target volumes | CTV_Ax | 0.69 (0.07) | 3.41 (1.13) | 0.63 (0.11) | 0.61 (0.18) | 4.04 (2.89) | 0.60 (0.18) |
|  |  | CTV_Chestwall | 0.67 (0.14) | 4.44 (2.21) | 0.69 (0.16) | 0.68 (0.15) | 4.37 (2.47) | 0.68 (0.19) |
|  |  | CTV_IMN | 0.47 (0.14) | 2.71 (1.48) | 0.72 (0.16) | 0.36 (0.26) | 4.29 (5.09) | 0.59 (0.30) |
|  |  | CTV_Sclav_LN | 0.57 (0.12) | 3.65 (1.58) | 0.65 (0.14) | 0.56 (0.21) | 3.82 (2.65) | 0.63 (0.21) |
|  | OARs | BrachialPlex_L | 0.20 (0.27) | 4.19 (15.64) | 0.58 (0.52) | 0.25 (0.20) | 7.23 (9.53) | 0.59 (0.29) |
|  |  | Heart | 0.91 (0.08) | 1.84 (1.39) | 0.74 (0.13) | 0.93 (0.07) | 1.49 (1.18) | 0.75 (0.17) |
|  |  | A_LAD | 0.33 (0.13) | 4.59 (2.93) | 0.72 (0.12) | 0.31 (0.30) | 6.20 (8.85) | 0.62 (0.40) |
| Sarcoma | Target volumes | GTV | 0.94 (0.02) | 0.39 (0.24) | 0.80 (0.09) | 0.92 (0.14) | 0.47 (1.11) | 0.69 (0.40) |
|  |  | CTV | 0.72 (0.16) | 5.36 (4.66) | 0.72 (0.23) | 0.67 (0.31) | 4.73 (6.43) | 0.64 (0.47) |
|  | OARs | Genitals | 0.69 (0.04) | 3.19 (0.58) | 0.73 (0.06) | 0.58 (0.31) | 4.27 (5.93) | 0.61 (0.32) |
| Head and Neck | Target volumes | GTVp | 0.79 (0.06) | 1.40 (0.77) | 0.68 (0.15) | 0.74 (0.12) | 2.35 (3.44) | 0.62 (0.20) |
|  |  | GTVn | 0.91 (0.02) | 0.52 (0.16) | 0.64 (0.07) | 0.87 (0.10) | 0.84 (3.34) | 0.53 (0.20) |
|  |  | CTV1 | 0.85 (0.07) | 1.32 (0.77) | 0.65 (0.16) | 0.62 (0.34) | 6.75 (9.16) | 0.35 (0.32) |
|  |  | CTV2 | 0.71 (0.29) | 10.35 (12.58) | 0.83 (0.36) | 0.40 (0.45) | 18.98 (37.41) | 0.64 (0.43) |
|  | OARs | Brainstem | 0.82 (0.24) | 1.08 (1.20) | 0.74 (0.33) | 0.75 (0.16) | 1.59 (1.27) | 0.61 (0.26) |
|  |  | Glnd_Submand_L | 0.86 (0.05) | 0.54 (0.24) | 0.65 (0.10) | 0.84 (0.15) | 0.65 (0.70) | 0.59 (0.24) |
|  |  | Glnd_Submand_R | 0.80 (0.12) | 1.03 (0.95) | 0.78 (0.15) | 0.71 (0.30) | 1.27 (1.19) | 0.68 (0.23) |
|  |  | Larynx | 0.60 (0.30) | 2.17 (2.03) | 0.65 (0.33) | 0.60 (0.24) | 2.73 (1.69) | 0.54 (0.27) |
|  |  | Musc_Constrict | 0.58 (0.14) | 2.12 (1.14) | 0.76 (0.11) | 0.45 (0.24) | 2.84 (2.44) | 0.65 (0.27) |
|  |  | Parotid_L | 0.86 (0.04) | 0.90 (0.45) | 0.51 (0.08) | 0.80 (0.09) | 1.49 (1.23) | 0.40 (0.13) |
|  |  | Parotid_R | 0.87 (0.03) | 0.79 (0.34) | 0.51 (0.07) | 0.82 (0.10) | 1.31 (1.33) | 0.43 (0.14) |
| Gynecologic | Target volumes | GTVn | 0.79 (0.05) | 0.93 (3.19) | 0.43 (0.09) | 0.79 (0.45) | 1.28 (12.72) | 0.46 (0.40) |
|  |  | CTVn_4500 | 0.72 (0.03) | 3.03 (0.35) | 0.75 (0.06) | 0.66 (0.17) | 3.58 (2.12) | 0.68 (0.16) |
|  |  | CTVp_4500 | 0.79 (0.14) | 3.80 (2.48) | 0.75 (0.23) | 0.77 (0.13) | 3.63 (3.09) | 0.72 (0.20) |
|  | OARs | Bowel_Small | 0.80 (0.14) | 5.04 (3.02) | 0.70 (0.11) | 0.57 (0.40) | 8.38 (7.08) | 0.52 (0.27) |
| Gastrointestinal | Target volumes | CTV_4500 | 0.76 (0.03) | 4.10 (0.40) | 0.71 (0.08) | 0.65 (0.21) | 4.75 (3.12) | 0.66 (0.16) |
|  |  | CTV_5400 | 0.63 (0.20) | 15.22 (23.40) | 0.79 (0.40) | 0.48 (0.34) | 6.84 (5.90) | 0.87 (0.28) |
|  | OARs | Bag_Bowel | 0.64 (0.08) | 6.13 (2.50) | 0.65 (0.08) | 0.59 (0.27) | 7.68 (6.43) | 0.60 (0.21) |
